# Compound heterozygous hemophilia A in a female patient and the identification of a novel missense mutation, p.Met1093Ile

**DOI:** 10.3892/mmr.2013.1841

**Published:** 2013-12-04

**Authors:** SHU-KAI QIAO, HAN-YUN REN, JIN-HAI REN, XIAO-NAN GUO

**Affiliations:** 1 Department of Hematology, Peking University First Hospital, Beijing 100034, P.R. China; 2 Department of Hematology, The Second Hospital of Hebei Medical University, Shijiazhuang, Hebei 050000, P.R. China

**Keywords:** factor VIII, hemophilia A, female, F8, mutation

## Abstract

Hemophilia A (HA) in females is rare. Female HA cases are often misdiagnosed as acquired HA (AHA) or as von Willebrand disease type 2N (vWD-2N). Here, we report the case of a 37-year-old female HA patient with a moderate factor VIII (FVIII) deficiency. The patient had no personal or family history of bleeding disorders, but presented with heavy uterine bleeding following surgery to remove an intrauterine device. IgG inhibitory antibodies against FVIII were undetected. A compound heterozygote mutation of the FVIII gene (F8) was found in this patient. The p.Val502Asp mutation, which has been reported previously, affects A2 domain function. A novel missense point mutation, p.Met1093Ile, was identified in the B domain. The compound heterozygote mutations in F8, p.Val502Asp and p.Met1093Ile, caused HA in this female patient, with a moderate phenotype.

## Introduction

Hemophilia A (HA) is an X-linked recessive congenital coagulation disorder caused by a factor VIII (FVIII) deficiency, which results from mutations in the FVIII gene (F8). HA typically occurs in males with an incidence of ~1 in 5000 (1). Female HA patients have rarely been reported, although they may carry a defective allele. Human F8 maps to the long arm of the Xq28 region of the X chromosome and consists of 26 exons and 25 introns. The protein product of F8 possesses no enzyme activity. The 19 amino acid signal peptide at the N-terminus, encoded by an open reading frame (ORF), leads to the passage of FVIII through hepatocytes to blood vessels (2). The matured FVIII protein is composed of 2332 amino acids and comprises three homologous A domains, two homologous C domains and a single B domain. These domains, arranged as A1-A2-B-A3-C1-C2 from the N- to the C-terminus, are important for FVIII function. FVIII synthesized in hepatocytes is secreted into the plasma in the form of an inactive pro-cofactor (3). FVIII, an essential cofactor for coagulation factor IX, readily binds to von Willebrand factor (vWF), forming a tight non-covalent complex that protects against degradation. Furthermore, FVIII may also interact with a number of proteins, such as thrombin and factor X. These interactions are indispensable for effective coagulation. Once FVIII is cleaved by thrombin, activated FVIII dissociates from vWF and participates in the coagulation cascade (4).

Genetic abnormalities in F8 may result in qualitative or quantitative defects of the FVIII protein. The most common F8 abnormality to cause HA is an inversion of intron 1 or 22 (5,6). The remaining HA cases are caused by numerous point mutations spread throughout the gene, including missense, nonsense, splice site, frameshift mutations and gross deletions (7,8). To date, >1400 F8 mutations within the FVIII coding and non-coding regions have been identified in the Haemophilia A Mutation, Search, Test and Resource Site (HAMSTeRS) database (http://hadb.org.uk/).

In this study, we report the case of a female HA patient with F8 compound heterozygote mutations. Diagnosis and differential diagnosis were complex. Furthermore, F8 compound heterozygote mutations (c.1505T>A, p.Val502Asp and c.3279G>A, p.Met1093Ile) were identified using multiplex PCR and DNA sequencing.

## Case report

### Patient history

A 37-year-old female patient presented with an 8-month history of severe uterine abnormal bleeding following surgery for the removal of an intrauterine device. After a short time, the patient developed profound anemia (hemoglobin level of 58 g/l). Prior to being admitted to our hospital, the patient had received a blood transfusion (1200 ml) for hemorrhagic anemia in the Second Hospital of Hebei Medical University. Furthermore, the patient complained of repeated skin purpura since childhood. The patient had no other history of bleeding, such as nosebleeds, gingival bleeding, hematemesis or melena. There was no personal or family history of bleeding disorders. The patient denied any intake of medicines or herbs. The biochemical and hematological data of the patient are shown in Table I.

White blood cell and platelet levels were within the normal ranges. Hemoglobin was only marginally reduced (92 g/l), due to the blood transfusion. The prothrombin time (PT) and international normalized ratio (INR) were normal, but the activated partial thromboplastin time (APTT) and bleeding time (BT) were noticeably prolonged, which may be corrected easily by a plasma transfusion. Plasma FVIII levels were significantly decreased (4.7%). vWF antigen levels were within the normal range (128%). Autoimmune markers, such as the antinuclear, anticardiolipin, antidouble stranded DNA and extractable nuclear antigen (ENA) polypeptide antibodies were all negative. Other coagulation tests were also normal, including platelet count and function, and fibrinogen levels.

### Diagnosis and differential diagnosis

Based on the clinical manifestations and laboratory tests, the following diagnoses were considered, in order of decreasing probability: i) Acquired HA (AHA); ii) von Willebrand disease type 2N (vWD-Type 2N); and iii) HA. There is a certain degree of overlap in the clinical manifestations and laboratory results among these three disorders. Although female HA is extremely rare, it could not be ruled out as a possibility. The key points of diagnosis and differential diagnosis for these three diseases are discussed in the following paragraphs.

AHA is an autoimmune disorder caused by autoantibodies against FVIII, neutralizing its coagulation functions and resulting in severe, often life-threatening bleeding. It is characterized by severe, spontaneous hemorrhaging at sites, such as the skin, muscle and soft tissues, or by excessive bleeding during surgery. Hemarthrosis, the hallmark of severe congenital HA, seldom occurs in AHA patients (9). AHA may be associated with several clinical conditions, including pregnancy, autoimmune diseases, malignancies, infections or drugs. Approximately 50% of patients are idiopathic with no known underlying disease association (10). If a patient presents with spontaneous hemorrhaging, then previous personal or family history of bleeding must be ruled out. Once laboratory results reveal the isolated prolongation of APTT and reduced FVIII levels, which fail to normalize following a normal plasma transfusion, AHA should be suspected. Following confirmation that FVIII antibodies are absent and that FVIII levels may be improved by transfusing normal plasma, a diagnosis of AHA was definitively ruled out.

vWD is the most common inherited bleeding disorder and is caused by deficiency or dysfunction of vWF. vWD is divided into three types: Type 1 (partial quantitative deficiency); Type 2, with four subtypes 2A, 2B, 2M and 2N (qualitative deficiency); and Type 3 (complete quantitative deficiency) (11). Type 2N vWD show a marked decrease in the vWF binding affinity for FVIII. Its clinical manifestations are similar to that of mild HA: FVIII levels are decreased, but vWF antigen (vWF:Ag) levels are within the normal range (12). These patients are usually misdiagnosed as HA. When the laboratory results were returned, it was observed that the vWF binding affinity for FVIII and the ristocetin-induced platelet agglutination (RIPA) were normal in our patient. Therefore, a diagnosis of vWD-Type 2N was ruled out. Thus, a diagnosis of HA was considered for the patient. The differential diagnoses of HA, VWD-Type 2N and AHA are outlined in Table II.

### Mutational screening

In order to confirm the cause of HA in the patient, a mutational screening of F8 was performed. Genomic DNA was obtained from EDTA-anticoagulated blood from the patient. Inversions in introns 1 and 22 were first screened using long-distance PCR (13). All exons and their flanking regions of F8 were amplified by PCR using specific primers, as described previously (14). PCR products were purified and sequenced.

### DNA sequencing

**Figure 1:**
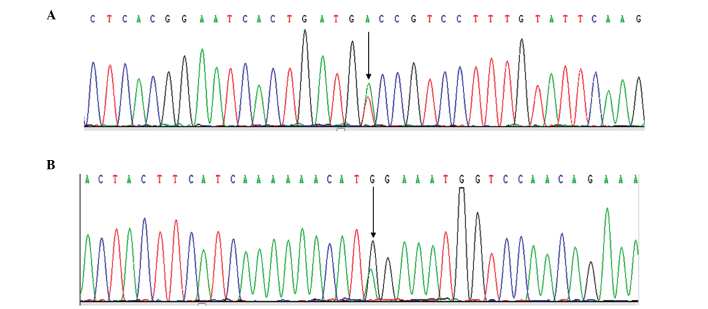
Results from DNA sequencing of exons 10 and 14. (A) Exon 10: NM_000132.3 c.1505T>A (p.Val502Asp). (B) Exon 14: NM_000132.3 c.3279G>A (p.Met1093Ile).

Introns 1 and 22 were not inverted. A previously reported mutation (15) was detected (exon 10, c.1505T>A, p.Val502Asp), and a novel unreported missense point mutation (exon 14, c.3279G>A, p.Met1093Ile) (Fig. 1).

### Molecular modeling

**Figure 2:**
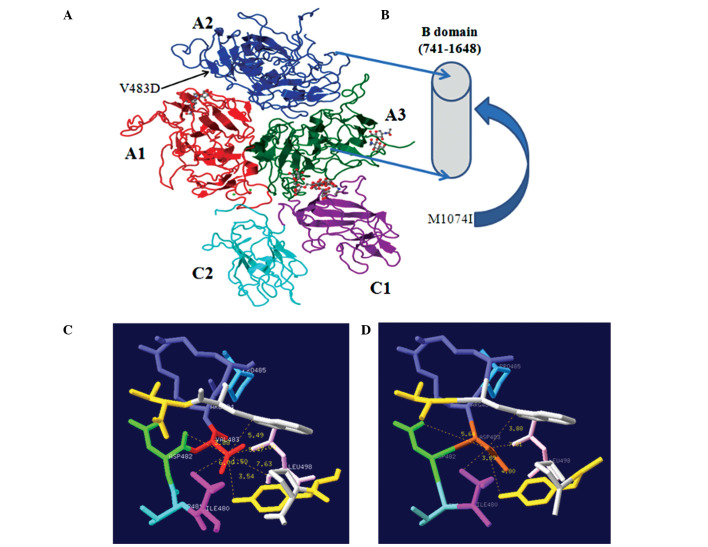
Modeled structure of compound heterozygote mutated FVIII protein. (A) Crystal structure of B domain-deleted FVIII (PDB ID 2R7E). The domains are individually labeled and colored. The p.Val502Asp mutation located in the A2 domain is indicated by an arrow. (B) Simple model of the FVIII B domain. The p.Met1093Ile mutation is located in the B domain and is indicated by an arrow. The B domain spans the sequence from amino acid 741 to 1648. Unlike A and C domains, no available molecular models or crystal structures helped to elucidate the detailed structure of the B domain. (C and D) Comparison of p.Val502Asp mutation structural images in A2 domain of FVIII generated using the Swiss-Pdb Viewer.

Mutated FVIII proteins were modeled using the crystal structure of activated recombinant FVIII (16,17). Structural images were generated using the Swiss-Pdb Viewer (Swiss Institute of Bioinformatics, Geneva, Switzerland) (18) (Fig. 2).

### Conserved domains analysis

A conserved domains analysis was conducted for p.Val502 and p.Met1093 by using the Multiple Alignment Tool (http://www.ncbi.nlm.nih.gov/tools/cobalt/cobalt.cgi?link_loc=BlastHomeLink). It was observed that p.Val502 of the A2 domains in F8 is highly conserved among mammals (Table III). By contrast, p.Met1093 in the B domain shares little amino acid homology, indicating that it is not conserved among different species.

## Discussion

Due to the X-linked recessive mode of inheritance, HA usually affects males, and females usually are carriers who may pass the disease on to their progeny (19). Thus, female HA cases are rarely observed. However, in certain cases, there are a variety of potential genetic mechanisms leading to HA in females: i) Non-random inactivation of the normal X-chromosome in a female HA carriers (the most common cause of female HA (20,21); ii) homozygous F8 mutations (mostly reported in India where consanguineous marriages are more common) (22,23); iii) compound heterozygous mutations affecting both F8 alleles (21); and iv) X-chromosome monosomy or gross structural defects, such as a deletion or translocation (24–26).

Disease severity in HA patients is classified according to residual plasma FVIII activity (FVIII:C): severe (<1%), moderate (1–5%) and mild (5–35%) (27). In the severe phenotype, the most prevalent mutations are inversions of introns 1 or 22, accounting for 5% and 40–50% of patients, respectively (5,28). Moderate or mild HA are usually caused by missense mutations (29).

In this study, we presented the case of a female HA patient with a moderately clinical phenotype (FVIII: C=4.8%) resulting from F8 compound heterozygous mutations. The p.Val502Asp mutation located in A2 domains was first reported to be associated with mild HA by Fernández-López *et al* (15) in 2005. As the original amino acid Val502 in F8 is highly conserved among all known mammals, it was predicted that Val502 is involved in an A2-specific functional role. The non-polar, hydrophobic amino acid Val is replaced by the polar and acidic amino acid Asp, resulting in altered stability and protein folding. The p.Met1093Ile mutation is located in the B domain of F8, which is encoded by exon 14 (amino acids 741–1648). Contrary to the A and C domains, there are no available molecular models or crystal structures that elucidate the detailed structural information of the B domain. The FVIII B domain shares little sequence homology with other known mammalian species. Despite the fact that the B domain is not directly required for FVIII coagulation activity, it has been shown to exhibit a major role in the intracellular interactions that regulate quality control and secretion, as well as potential regulatory roles within plasma during activation, platelet binding, inactivation and clearance (30). Therefore, the p.Met1093Ile mutation in the B domain also affects FVIII coagulation activity. Furthermore, missense mutations within the F8 B domain have often been reported in HA patients (31,32). p.Met1093Ile is a novel missense mutation unreported in the HAMSTeRS database. The mutation was submitted to the HAMSTeRS database and was accepted (unpublished). It was estimated that the compound heterozygous mutations (p.Val502Asp and p.Met1093Ile) are causative gene defects, which resulted in a moderate HA phenotype in this female patient.

In conclusion, female HA patients, particularly those without a personal or family history of bleeding disorders, are often misdiagnosed as AHA or vWD-type 2N. A diagnosis of HA should be made in a female patient when isolated APTT is prolonged, FVIII binding capacity for vWF is normal, FVIII levels are decreased and FVIII auto-antibodies are absent. Genetic analysis of F8 using multiplex PCR and DNA sequencing is an essential tool in elucidating the nature of the various molecular mechanisms resulting in HA in females.

## Figures and Tables

**Table I:** Initial biochemistry and hematology results.

Variable	Value	Reference range
Whole blood		
White blood cell	5.8×10^9^/l	type4/xref>–10)x10^9^/l
Hemoglobin	92 g/l	110–155 g/l
Platelet	216×10^9^/l	(100–300)×10^9^/l
Coagulation panel		
PT	14.6 sec	12–16 sec
INR	1.12	0.8–1.2
APTT	46.5 sec	24–37sec
APTT-R	1.62	0.8–1.2
Fib	242 mg/dl	200–400 mg/dl
BT	11 min	4.8–9 min
FVIII:C	4.7%	50–150%
vWF-Ag	128%	60–150%
LA	29.5 sec	27–41 sec
RIPA (1.2 mg/ml)	91%	87–102%
Factor VIII antibody	Negative	Negative

PT, prothrombin time; INR, international normalized ratio; APTT: activated partial thromboplastin time; Fib, fibrinogen; BT, bleeding time; vWF-Ag, von Willebrand factor antigen; LA, lupus anticoagulant; RIPA, ristocetin induced platelet agglutination.

**Table II:** Differential diagnosis of HA, vWD-Type 2N and AHA.

Variable	HA	vWD-Type 2N	AHA
BT	Normal	Normal or (↑)	Normal
APTT	↑	↑	↑
FVIII:C	↓	↓	↓
vWF-Ag	Normal	(↓) or Normal	Normal
FVIII inhibitor	Negative	Negative	Positive
vWF-FVIII binding affinity	Normal	↓	Normal
vWF:Rcof	Normal	(↓) or Normal	Normal

HA: hemophilia A; AHA: Acquired HA; vWD, von Willebrand disease; FVIII:C, coagulation factor VIII activity; vWF, von Willebrand factor; vWF:Ag, vWF antigen; vWF:RCof, vWF ristocetin cofactor activity.

**Table III:** Conserved domains analysis in F8 for p.V483(502).

Species	Domain	Nucleotide number
Hs	PLLYGEVGDTLLIIFKNQASRPYNIYPHGITD**V**RPLYSRRLPKGVKHLKDFPILGEIFKYKWTVTVEDGPT	541
Mm	PLLYGEVGDTLLIIFKNQASRPYNIYPHGITD**V**SPLHARRLPRGIKHVKDLPIHPGEIFKYKWTVTVEDGPT	541
Rn	PLLYGEVGDSLLIVFKNRASRAYNIHPHGIRD**V**GAVHAGRLPRGVKHVKDLPIRPGETFKYRWTLTAEDGPA	529
Cf	PLLYGEVGDTLLIIFKKQASRPYNIYPHGINY**V**TPLHTGRLPKGVKHLKDMPILPGEIFKYKWTVTVEDGPT	535
Bt	PLLYGEVGDTLLIIFKNQASRPYNIYPHGITD**V**SPLHSGRFPKGVKHLKDMPILGEVFKYKWTVTVEDGPT	536
Oc	PLLYGEVGDTLLIIFKNQASRPYNIYPHGITD**V**SPLHSGRLSKGMKHLKDLPILPGEIFYKWKVTVEDGPT	537
Gg	PVLKGEVGDQFKIVFRNLASRPYNIYPHGLTS**V**NPYHAMKPSQGKKDVKDIPAPGQSFTYRWSITTEDGPT	529
Oa	PLLYGEVGDTLLIIFKNQASRPYNIYPHGITD**V**SPLHSGRFPKGVKHLKDMPILPGEVFKYKWTVTVEDGPT	536
Ss	PLLYGEVGDTLLIIFKNKASRPYNIYPHGITD**V**SALHPGRLLKGWKHLKDMPILPGETFKYKWTVTVEDGPT	541
Tr	PLLKGKVGDQIHIMLKNTASRPFNIYPNGLSS**I**RPMKRSKNAS-EKDLRTMGVGPNETFGYMWELTANDRPL	546
Sh	PLLYGEVGDMLLITFKNLASRPYNIYPHGLTS**V**SPLHSGRLPKGVKDVKDMPIMPGQTFKYKWEVTMEDGPT	541
La	PLLYGEVGDTLLIIFKNQASRPYNIYPHGITN**V**SPLHSGRLSKGVKHLKDLQIMPGEIFKYKWTVTLEDGPT	542
Dr	PELRGEVGDKFQIVFKNMASRPFNIYPNGLTS**V**QPLKTTNKDK-QVDLRSLAVPPGEIMTYLWKLTAEGDPT	530

V483(502) is highly conserved among mammalians (in bold). Hs, *Homo sapiens*; Mm, *Mus musculus*; Rn, *Rattus norvegicus*; Cf, *Canis lupus familiaris*; Bt, *Bos taurus*; Oc, *Oryctolagus cuniculus*; Gg, *Gallus gallus*; Oa, *Ovis aries*; Ss, *Sus scrofa*; Tr, *Takifugu rubripes*; Sh, *Sarcophilus harrisii*; La, *Loxodonta africana*; Dr, *Danio rerio*.
